# Condensin HEAT Subunits Required for DNA Repair, Kinetochore/Centromere Function and Ploidy Maintenance in Fission Yeast

**DOI:** 10.1371/journal.pone.0119347

**Published:** 2015-03-12

**Authors:** Xingya Xu, Norihiko Nakazawa, Mitsuhiro Yanagida

**Affiliations:** 1 Okinawa Institute of Science and Technology Graduate University, Onna-son, Okinawa, Japan; 2 Department of Genetics, Graduate School of Medicine, Osaka University, Suita, Osaka, Japan; University of Oklahoma, UNITED STATES

## Abstract

Condensin, a central player in eukaryotic chromosomal dynamics, contains five evolutionarily-conserved subunits. Two SMC (structural maintenance of chromosomes) subunits contain ATPase, hinge, and coiled-coil domains. One non-SMC subunit is similar to bacterial kleisin, and two other non-SMC subunits contain HEAT (similar to armadillo) repeats. Here we report isolation and characterization of 21 fission yeast (*Schizosaccharomyces pombe*) mutants for three non-SMC subunits, created using error-prone mutagenesis that resulted in single-amino acid substitutions. Beside condensation, segregation, and DNA repair defects, similar to those observed in previously isolated SMC and *cnd2* mutants, novel phenotypes were observed for mutants of HEAT-repeats containing Cnd1 and Cnd3 subunits. *cnd3-L269P* is hypersensitive to the microtubule poison, thiabendazole, revealing defects in kinetochore/centromere and spindle assembly checkpoints. Three *cnd1* and three *cnd3* mutants increased cell size and doubled DNA content, thereby eliminating the haploid state. Five of these mutations reside in helix B of HEAT repeats. Two non-SMC condensin subunits, Cnd1 and Cnd3, are thus implicated in ploidy maintenance.

## Introduction

Condensin plays a major role in chromosome dynamics during mitosis, interphase, and development [1–4]. It is essential for chromosome condensation and segregation in diverse organisms, from bacteria and fungi, such as budding yeast and fission yeast, to flies and humans [5–15]. Condensin is also essential for DNA repair, such as excision repairs that remove DNA lesions, dosage compensation, and development in higher eukaryotes [16–22].

**Fig 1:**
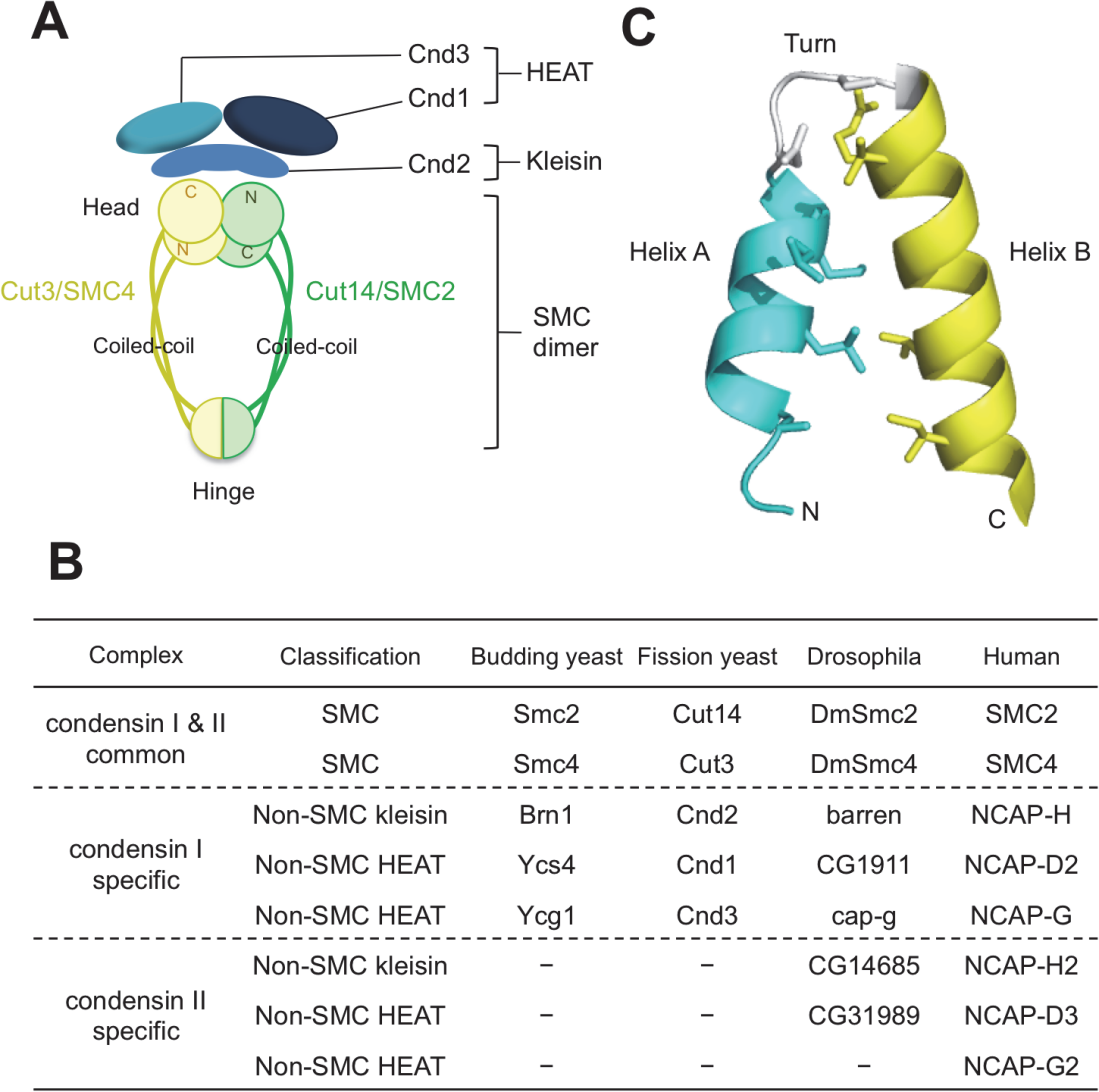
Nomenclature and structure of condensin subunits. **A**. Schematic presentation of fission yeast heteropentameric condensin subunits. Two of them (Cut3 and Cut14) belong to the family of SMC (structural maintenance of chromosomes) proteins, and three of them (Cnd1, Cnd2 and Cnd3) are non-SMC regulatory subunits. **B**. Table of SMC subunits and non-SMC subunits for budding yeast, fission yeast, *Drosophila*, and humans. Higher eukaryotes possess two kinds of condensin having the same SMC subunits, but distinct non-SMC subunits. **C**. Two non-SMC subunits (Cnd1 and Cnd3 in fission yeast) contain HEAT repeats, which contain two helices, A and B, connected with a turn. A representative HEAT unit is shown [34, 41].

Condensin contains five essential subunits that are conserved in eukaryotes. Two of them belong to the family of SMC (structural maintenance of chromosomes) proteins, which contain the terminal ATPase domains and the middle hinge domain, interrupted by long coiled coils (Fig. 1A) [14, 23, 24]. Six SMC proteins are known in eukaryotes, forming three distinct protein complexes, cohesin, condensin, and the SMC5-SMC6 complex [25, 26]. In the budding yeast, *Saccharomyces cerevisiae*, two condensin SMC proteins are called SMC2 and SMC4 [3, 14]. In the fission yeast, *Schizosaccharomyces pombe*, used in this study, Cut14 and Cut3 are, similar to SMC2 and SMC4, respectively [12]. Condensin has three additional non-SMC subunits (Fig. 1B). One of them has a sequence motif similar to that of bacterial kleisin, which interacts with the ATPase domains of SMC subunits [27–29]. Kleisin-like condensin subunits are present from bacteria to humans [30–35]. Two other non-SMC condensin subunits are present in budding yeast, and fission yeast, respectively [29, 30]. These show no resemblance to any cohesin subunits. In humans, non-SMC subunit composition is more complex than in fungi, because higher eukaryotes contain two classes of condensin (I and II) [2, 31]. Condensins I and II employ the same SMC subunits so that the principal differences between condensins I and II necessarily reside in the non-SMC subunits. Hence, understanding the roles of non-SMC subunits, which are largely unknown, even in fungi, is important for understanding diverse cellular functions of condensin.

HEAT repeats contain 30–40 amino acids and form two-folded helices, A and B, connected by a turn (Fig. 1C) [32, 33]. HEAT repeats are found in various cellular proteins involved in a variety of cellular functions. HEAT is an acronym designating four representative proteins, **H**untingtin, a protein that is linked to Huntington neurodegenerative disease, elongation factor 3 (**E**F3), involved in protein translation, protein phosphatase 2A (PP2A) regulatory subunit **A**, and the target of rapamycin kinase, **T**OR. In addition to these four proteins, importin β, implicated in protein transport [34, 35], ATM (Ataxia telangiectasia/Tel1) kinase, required for DNA damage sensing [36, 37], and XMAP215/Dis1/TOG, implicated in chromosome segregation and kinetochore function [38–40], contain HEAT repeats. Together, these proteins comprise an impressive group of important cell signaling and regulatory proteins. While the exact role of HEAT repeats is scarcely understood, they are likely to be involved in protein-protein interactions [38, 41]. *S*. *pombe* condensin non-SMC subunits, Cnd1 and Cnd3, contain 7 and 9 HEAT repeats, respectively [41]. Armadillo (ARM) repeat proteins are structurally related to proteins containing tandem HEAT motifs, and the two protein families probably have a common phylogenetic origin [42, 43].

In this study, we attempted to construct mutants of fission yeast condensin non-SMC subunits. We introduced mutations in the genomic *cnd* ^+^ genes by chromosomal integration of mutagenized gene fragments. Candidate integrant strains obtained by transformation were screened for different phenotypes, such as the temperature-sensitive (ts) phenotype or the phenotype hypersensitive to DNA damaging agents such as hydroxyurea (replication inhibitor inducing DNA strand breakage), camptothecin (inhibitor of DNA topoisomerase I), and UV irradiation. Mutation sites were determined by nucleotide sequence determination. To prove that the determined mutation sequences really caused the mutant phenotypes, we also introduced the mutated sequence into the wild-type genome to see whether the same phenotypes appeared in mutated wild-type yeast. We were thus able to obtain and characterize 21 newly isolated mutants of *cnd1*, *cnd2*, and *cnd3*, containing only single amino acid substitutions.

The novelty of our approach for isolating condensin non-SMC mutants was that we did not screen the microscopic condensation-defect, which was used as the selective phenotype in previous studies [17, 44]. In this study we isolated mutants that produced ts and/or DNA damage-sensitive phenotypes.

## Results

### Condensin non-SMC mutants produced by error-prone mutagenesis

**Fig 2:**
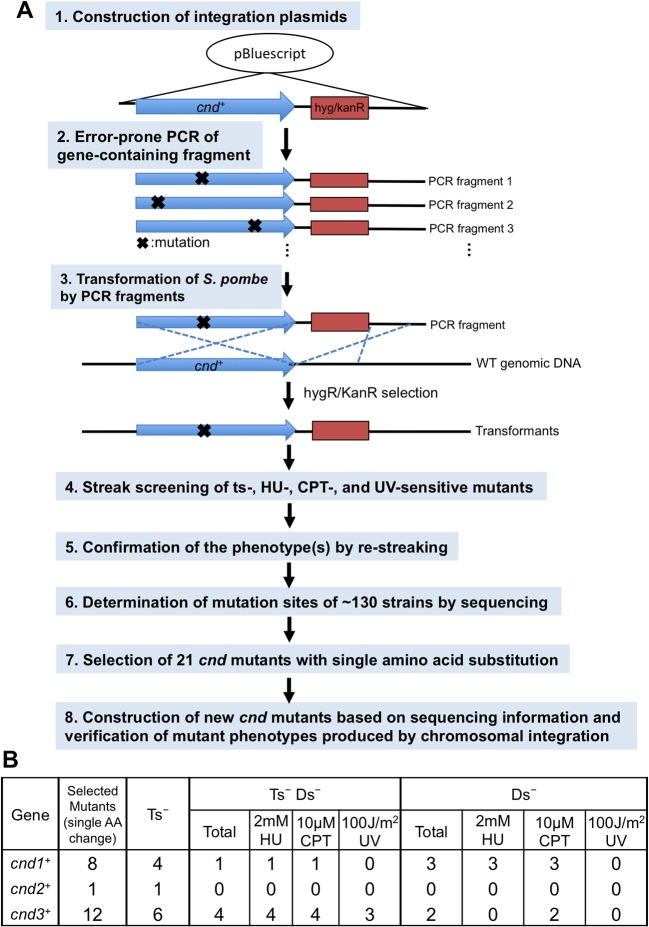
Screening procedures for non-SMC subunit mutants of *S*. *pombe* condensin. **A**. Procedures consist of eight steps, starting from construction of integration plasmids and ending with verification of the *cnd* mutant phenotype produced by chromosomal re-integration (see text). **B**. Twenty-one temperature- and/or DNA damage-sensitive *cnd* mutant strains containing various single amino acid substitutions were obtained. Their classification according to phenotype is shown in the table (see text). Ts^-^, temperature sensitive; Ds^-^, damage sensitive; Ts^-^Ds^-^, temperature and damage sensitive.

Temperature-sensitive (ts) *S*. *pombe* condensin SMC and non-SMC mutants that exhibited chromosome segregation and condensation defects were previously isolated by cytological screening of many *ts* strains [12, 16, 17, 44–46]. Among them, *cut14 (smc2)-Y1* and *cnd2 (kleisin)-1* were sensitive to DNA damage [16, 17]. Genetic dissections of condensin’s non-SMC subunit functions have been scarce. To characterize these subunits in a systematic way, we isolated mutants for all 3 subunits using error-prone mutagenesis [47] (Fig. 2A step1-8). Three plasmids (pBS-CND1, -CND2, and -CND3, Materials and Methods), carrying one of the three *cnd* ^+^ genes, with the hygR/kanR marker and 500 bp downstream sequences (described in Materials and Methods), were constructed and employed in PCR mutagenesis (using a high magnesium concentration, 8 mM). Mutations were introduced at the rate of approximately one nucleotide per kb (estimated by nucleotide sequence determination).

Then mutagenized DNA fragments were used for transformation of *S*. *pombe* by chromosomal replacement integration through homologous recombination. Resulting transformants with antibiotic drug resistance markers were plated, and properties of colony formation were manually determined by streaking (in total ∼18,000 transformant colonies) on agar plates. In addition, each colony was streaked under five different culture conditions: 1) standard culture conditions (26°C without any additional treatment), 2) at 36°C (to examine the ts phenotype through the lack of colony formation at 36°C, the restrictive temperature, for 3 days), 3) 2 mM hydroxyurea (HU) at 26°C (sensitivity to DNA replication inhibitor drug at the permissive temperature), 4) 7.5 μM camptothecin (CPT) at 26°C (sensitivity to type 1 topoisomerase poison), 5) 150 J/m^2^ ultraviolet ray (UV) irradiation at 26°C (sensitivity to UV irradiation). Candidate Ts^-^ and/or damage sensitive (Ds^-^) colonies were re-streaked to confirm the phenotypes (Step 5 in Fig. 2A).

### Characterization of isolated mutants

We obtained 41, 10, and 26 mutant strains, respectively, from transformants using mutagenized DNA fragments *cnd1*, *cnd2* and *cnd3* (S1 Table). Basically three kinds of mutant phenotypes, Ts^-^ only, both Ts^-^ Ds^-^, and Ds^-^ only, were obtained (Ds^-^ represents single or multiple sensitivities to HU, CPT or UV at 26°C). For *cnd1* mutants, 23 Ts^-^, 7 Ts^-^ Ds^-^, and 11 Ds^-^ were obtained, while 3 Ts^-^, 6 Ts^-^ Ds^-^, and 1 Ds^-^ were isolated for *cnd2*. For *cnd3* mutants, 8 Ts^-^, 12 Ts^-^Ds^-^, and 6 Ds^-^ were obtained. We then determined nucleotide sequences of *cnd* mutant genes for these 77 integrated mutants after PCR amplification. The majority (56/77) of strains contained multiple (2∼7) non-synonymous mutations in the *cnd1*, *cnd2*, and *cnd3* genes, so they were not investigated further. The remaining 21 strains (8 *cnd1*, 1 *cnd2*, 12 *cnd3*), containing only single non-synonymous mutations were selected for further study (Step 7 in Fig. 2A).

To verify that the sequence change in each mutant really caused the phenotype, we then newly constructed *cnd* mutants based on sequencing information by re-integrating the linearized plasmids containing one of the 21 mutant sequences onto the wild-type *S*. *pombe* genome. All of the resulting transformants containing chromosomal gene replacements with mutant genes produced the expected phenotypes (Step 8 in Fig. 2A). Further tetrad dissection was conducted to prove that the ts mutation was really caused by a mutation at one locus. The Ts^+^:Ts^-^ markers from all mutants segregated 2:2, and the ts phenotype was tightly linked to the antibiotic drug-resistant phenotype. Hence, we concluded that these 21 newly isolated mutants, containing single amino acid substitutions, could cause either Ts^-^, Ts^-^ Ds^-^, or Ds^-^ phenotypes (Fig. 2B).

**Fig 3:**
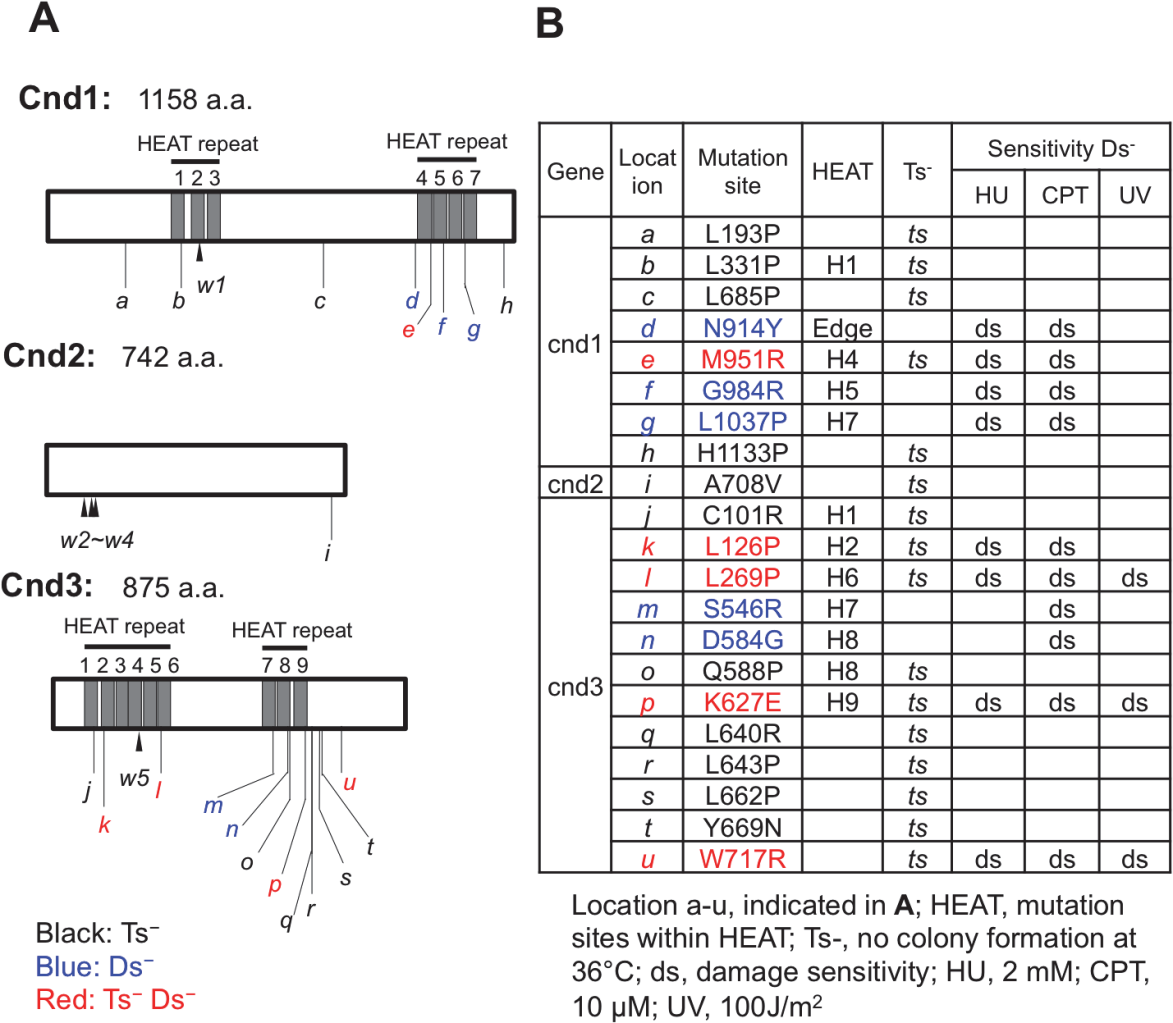
Location of mutation sites and phenotypes of twenty-one *cnd1*, *cnd2* and *cnd3* integrants. **A**. Location of mutation sites determined by nucleotide sequencing. a-u show 21 mutants, indicated in Fig. 3B. Black, blue, and red letters represent Ts^-^, Ds^-^, and Ts^-^Ds^-^, respectively. Location of previously isolated mutants, w1-w3 and w5 [44], w4 [17] are shown. **B**. Mutational changes of amino acids and locations in HEAT repeats (H with number, indicated in a). Temperature-sensitivity and damage-sensitivity of each mutant are also shown. The mutation of *cnd1-N914Y* is located near the edge of HEAT repeat 4 (indicated as Edge).

Locations of newly identified mutations in the *cnd1*, *2*, and *3* genes are shown in Fig. 3A (a-u), together with information regarding the substitution mutant sequences (Table in Fig. 3B). Locations of the 5 previously isolated non-SMC ts mutants are shown as w1-w5 (explained in the figure legend). In these newly identified 21 mutants, 11 (4 *cnd1*, 1 *cnd2*, and 6 *cnd3*) displayed only the ts phenotypes (black characters). Five (red characters in Fig. 3) showed both ts (36°C) and damage sensitive phenotypes (at 26°C, phenotypic markers are indicated as Ts^-^ Ds^-^). The remaining five mutants (3 *cnd1* and 2 *cnd3*) are damage-sensitive and not ts. Among the 21 strains, 11 mutations reside within the HEAT repeats (indicated by H and HEAT repeat number in the repeat clusters). One mutant, *cnd1-N914Y*, is located near the edge of HEAT repeat 4 (indicated as Edge in Fig. 3B). Note that many amino acid substitution mutations (10 of 21) introduce proline (P), which is known as a helix breaker. In addition, glycine (G) also disrupts helices. Arginine residues (R), which possess a bulky side chain, are also plentiful (6 of 21). The significance of these substitutions from wild-type amino acids (9 of 21 are L) to P and R is discussed below, with their implications for the secondary structure of HEAT repeats.

### Defects in chromosome segregation caused by Ts^-^ mutations

**Fig 4:**
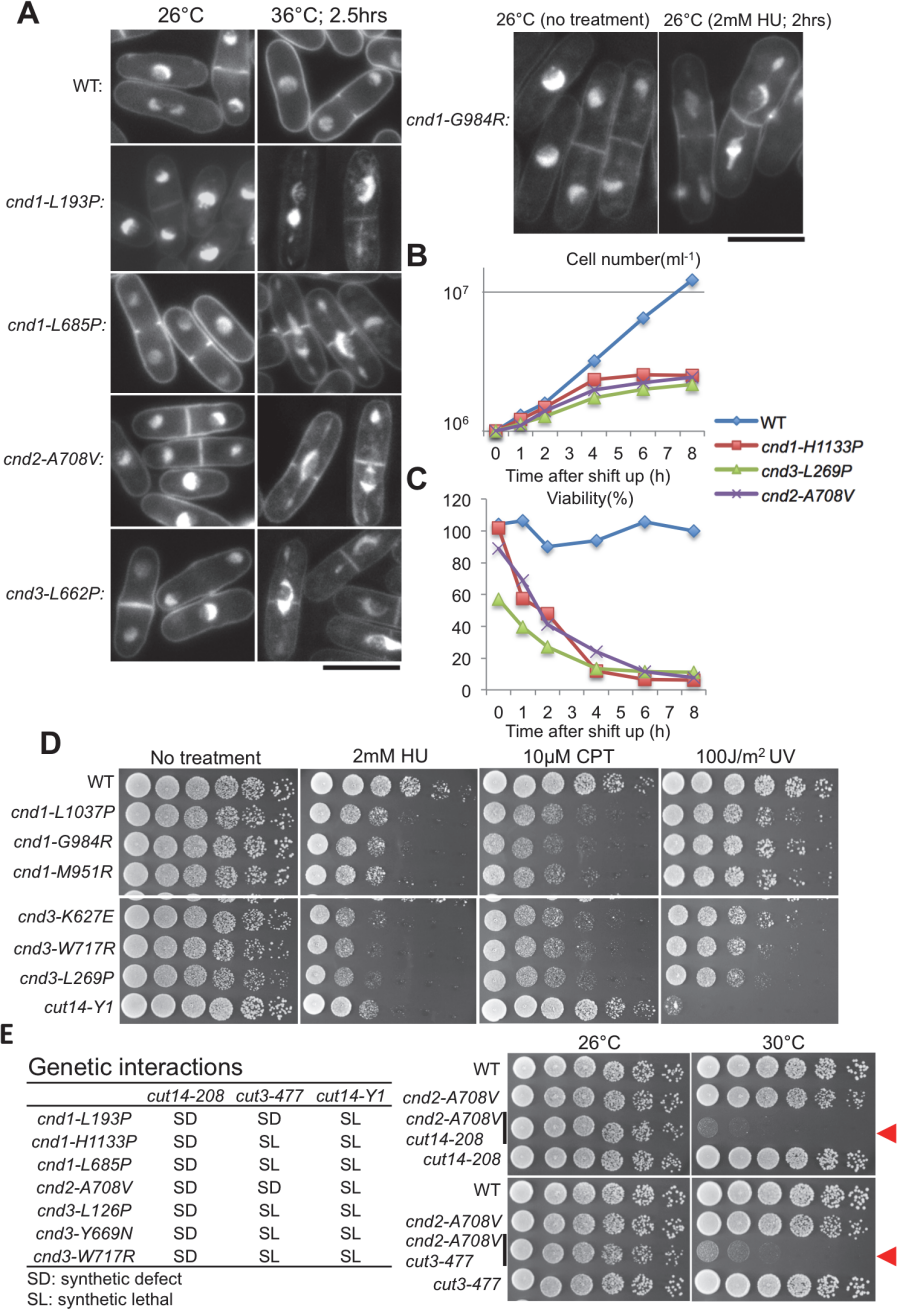
Phenotypes of cytological, drug, and synthetic genetic interaction phenotypes of mutants. **A**. DAPI-stained *S*. *pombe* wild-type (WT) and mutants cells cultured at 26°C and 36°C for 2.5 h. Cells displaying severe defects in chromosome segregation were observed in mutant cells at 36°C. More than 100 mitotic cells for each mutant strain were counted. Almost all (95∼100%) mitotic mutant cells observed after 2.5 hrs at 36°C showed severe defects of chromosome segregation as shown here. Mutant cells (*cnd1-G984R*) cultured in 2 mM HU for 2 h, which showed severe segregation defects at 26°C, are also shown (see text). Bars are 10 μm. **B** and **C**. In YPD liquid medium at the restrictive temperature, 36°C, ts mutants ceased to divide within 4 h and lost viability. Asynchronous cultures of WT and ts mutants (*cnd1-H1133P*, *cnd2-A708V* and *cnd3-L269P*) in the YPD liquid medium were shifted from 26°C to 36°C. Cell density (per mL) was determined and viability was measured after a temperature shift to 36°C. **D**. *S*. *pombe* wild-type (WT), and *cnd1* and *cnd3* mutant cells were spotted on solid agar plates in the presence or absence of HU, CPT, and UV irradiation. **E**. Synthetic genetic interactions between non-SMC ts mutants and SMC mutants (*cut14-208*, *cut3-477* and *cut14-Y1*). The left table summarizes all genetic interactions examined. The right panel shows the synthetic defect between *cnd2-A708V* and 2 SMC mutants (*cut14-208* and *cut3-477*). See text.

In liquid cultures, the above 16 ts mutants (11 Ts^-^ only and 5 Ts^-^ Ds^-^) were cultured first at 26°C and then shifted to 36°C for 0–8 hr. Cells were stained with DAPI (4,6-diamidino-2-phenylindole), a fluorescent probe for DNA, and were observed under a fluorescence microscope. Mutant cells collected from cultures after 2–4 h at 36°C showed severe defects in chromosome segregation. Micrographs from *cnd1-L193P* (a in Fig. 3A), -*L685P* (c), *cnd2-A708V* (i) and c*nd3-L662P* (s) show defects in condensation and segregation of mitotic chromosomes (Fig. 4A for 2.5 hrs at 36°C; S1 Fig. for 5 hrs at 36°C). ts *cnd1*, *cnd2*, and *cnd3* mutants examined so far at 36°C in liquid cultures, showed that defects in mitotic chromosome segregation revealed by DAPI staining were indistinguishable from those found in previously isolated ts condensin mutants of *S*. *pombe* [12, 16, 17]. phi-shaped nuclear chromatin is the hallmark of condensation deficiency. Damage-sensitive *cnd1-G984R* that was not ts was cultured at 26°C in the presence or absence of 2 mM HU (right panel in Fig. 4A). In the presence of HU, basically identical condensation and segregation defects, as in ts mutants, were observed. In the absence of HU (no treatment), no defects were observed.

Cell viability (%) and cell density (cells/mL) of cultures were measured for *cnd1-H1133P*, *cnd2-A708V*, and *cnd3-L269P* mutants by spreading cells on YPD plates and incubating them at 26°C for 4 days (Fig. 4B and C). Temperature sensitivity (spot assay) of these mutants was shown in S2 Fig. Mutant cells ceased to divide within 4 h at 36°C, the restrictive temperature. Viability decreased greatly after 4 h, consistent with the notion that loss of viability and cessation of cell division occurred after the failure of chromosome segregation.

### Many damage sensitive mutations reside in HEAT repeats

Among the 8 *cnd1* mutants, 4 mutations reside in the HEAT repeats (Fig. 3A). Three of the 4 are damage-sensitive. The other damage-sensitive mutant *cnd1-N914Y* is in close proximity to the C-terminal HEAT repeat 4. *cnd1-L1037P* (g in Fig. 3A), -*G984R* (f) and -*M951R* (e) are sensitive to HU and CPT, but not to UV (Fig. 4D).

Among the 12 *cnd3* mutants isolated, seven are located in HEAT repeats. All five damage-sensitive mutants are located in HEAT repeats (Fig. 3A). In Fig. 4D, sensitivities of *cnd3-K627E* (p), -*W717R* (u) and -*L269P* (l) to HU, CPT and UV are shown. In total, 11 *cnd1* and *cnd3* mutants are located in HEAT repeats. Among them, 8 are damage-sensitive. In contrast, for the 10 ts mutants (Ts^-^), which are not damage-sensitive, only 3 (3/10) are located in HEAT repeats. Interestingly, *cnd3* mutants are broadly sensitive to damage, as they were sensitive to UV as well as to HU and CPT. UV damage repair requires excision of thymine dimers formed at the lesion followed by homologous DNA recombination and gap-filling replication [48, 49].

### Genetic interactions of non-SMC subunit mutants with SMC mutants

To understand the role of non-SMC subunits in the condensin complex, genetic interactions between non-SMC and SMC mutants were examined (Fig. 4E). All 7 mutants (3 of *cnd1*, 1 of *cnd2*, and 3 of *cnd3*) caused synthetic defects (SD, poor colony formation) with *cut14-208* (S861P, mutated in the coiled coil). Two of them were synthetic defects involving *cut3-477* (S1147P, mutated in the coiled coil); the other 5, as shown using tetrad analysis, failed to produce double mutants (designated “synthetic lethal SL”). None of them can produce double mutants when combined with *cut14-Y1* (the mutation site of *cut14-Y1* is L543S). These data strongly suggest that non-SMC subunits work in a cooperative or parallel fashion with SMC subunits to form the condensin complex and to execute its functions. Results are consistent with the finding that double mutants *cnd2-A708V cut14-208* and *cnd2-A708V cut3-477* hardly formed colonies at the semi-restrictive temperature (30°C) (Fig. 4E, right panel) (red arrowheads). Newly isolated *cnd2-A708V* located at the C-terminus is ts, but not damage-sensitive, in contrast to the previously isolated N-terminal mutation *cnd2-1*, which is sensitive to DNA damage [17].

### Defective kinetochore segregation in the *cnd3-L269P* mutant, which is hypersensitive to a tubulin poison

**Fig 5:**
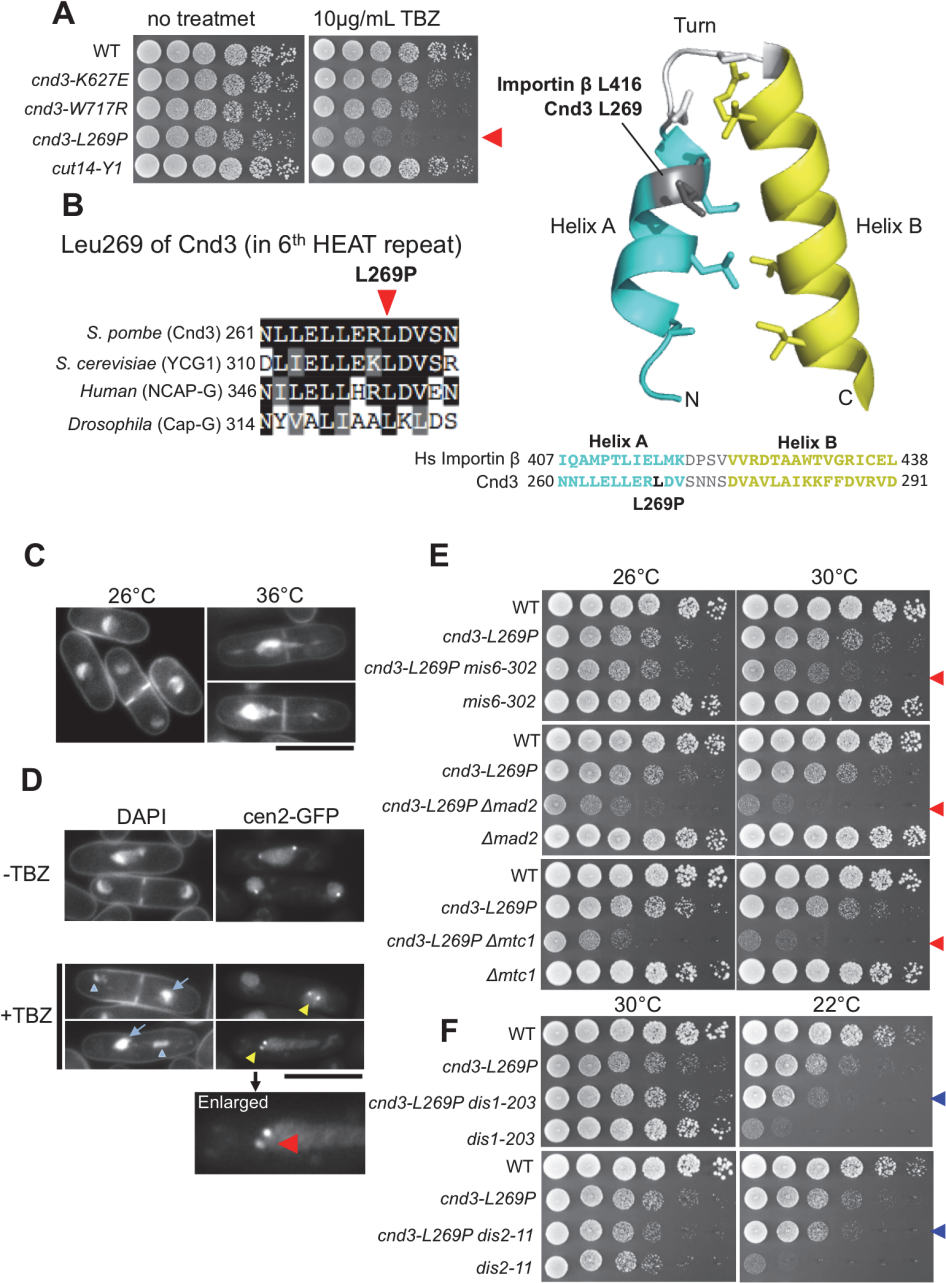
*cnd3-L269P*, a HEAT domain mutant is TBZ-sensitive and defective in kinetochore segregation. **A**. Only *cnd3-L269P* among 21 mutants is sensitive to TBZ. **B**. (left) The leucine residue (indicated by the arrowhead) is conserved among homologs from yeast to humans. (right) The mutation site is shown in the structure of a HEAT repeat determined for human importin β [34]. In HEAT, Cnd3 L269 corresponds to the position of importin β L416 residue located in helix A of the HEAT unit. The change to a helix-breaking proline strongly perturbs the helical structure. **C**. Mutant *cnd3-L269P* cells show severe mitotic defects at 36°C in the absence of TBZ. The bar is 10 μm. **D**. In the presence of TBZ, defective chromosome segregation seen as unequal segregation (2:0) of cen2 dots instead of equal segregation (1:1) [51] was observed at 26°C. Large and small daughter nuclei were indicated by light blue arrows and arrowheads, respectively. The bar is 10 μm. Enlarged micrograph for cen2-GFP unequal separation, indicated by the red arrowhead. Dot-like intense cen2-GFP signals were obtained by association of LacI repressor-GFP protein with the LacO operon repeats present in the cen2 region [57]. LacI-GFP containing nuclear localization signal is preferentially localized in the nucleus when it is unbound to the centromere. **E**. Synthetic genetic interactions of *cnd3-L269P* with kinetochore mutant, *mis6-302*, spindle checkpoint mutant, *Δmad2*, and XMAP215-like microtubule-interacting protein mutant, *Δmtc1/alp14*. **F**. Cold sensitivity of *dis1-*203 and *dis2-11* mutants was rescued when they were combined with *cnd3-L269P* mutation. See text.

We found that one of the *cnd3* Ts^-^ Ds^-^ mutants, L269P, with a mutation in the N-terminal (6^th^) HEAT repeat, was sensitive to a tubulin poison (10 μg/ml TBZ) (Fig. 5A). Amino acid sequence comparison indicated that residue L269 (gray color residue in the right panel of Fig. 5B) is highly conserved among homologs from other organisms, such as fungi, flies, and humans. L269 is present in helix A [34] of the 6^th^ HEAT repeat so that the introduction of a helix-breaking proline residue instead of leucine into the helix should cause significant structural perturbation. TBZ sensitivity was found only for *cnd3-L269P* among 21 condensin mutants isolated.

We observed typical condensin mutant defects in chromosome segregation producing phi (φ)-shaped chromosomes in *cnd3-L269P* mutants at 36°C, the restrictive temperature, in the absence of TBZ (Fig. 5C). No defect was found at 26°C. On the other hand, *cnd3-L269P* mutants cultured at 26°C, the permissive temperature, for 4 h in the presence of TBZ (added to the medium to a final concentration of 10 μg/mL) produced severe segregation defects (Fig. 5D, +TBZ). Mutant cells stained with DAPI revealed large (indicated by light blue arrow) and small (light blue arrowhead) daughter nuclei in a high frequency (45/68 mitotic cells), which are a typical phenotype of centromere mutants, like *mis6-302* and *mis12-537* [50]. In this mutant strain, the cen2-GFP probe used previously [51] was introduced (described in the legend of Fig. 5D). Cen2-GFP visualization further showed a high frequency (7/20 mitotic cells) of cen2 mis-segregation (2:0 rather than 1:1), in which two sister centromere GFP dots went into the same daughter cell (Fig. 5D, yellow arrowheads and a red arrowhead in the enlarged micrograph). In the absence of TBZ, large-small and 2:0 missegregation was hardly observed.

In addition to centromere mis-segregation, we found synthetic genetic interactions between *cnd3-L269P* and *mis6-302* (a centromere/kinetochore mutant [50, 52]) (Fig. 5E). Mis6 encodes a centromere protein essential for equal chromosome segregation. The synthetic lethal phenotype was also obtained with *Δmad2* deletion mutant [53]. Mad2 is an essential component for the spindle assembly checkpoint, SAC [54, 55]. The double mutant between *cnd3-L269P* and Δ*mad2* was lethal at both the permissive and semi-permissive temperatures (26–30°C). Note that the Δ*mtc1* deletion mutant implicated in mitotic spindle microtubule organization, was also additive with the defect caused by *cnd3-L269P*, as the double mutant hardly formed colonies at 26° and 30°C [40, 56]. Together, these results strongly suggest that the role of Cnd3 L269 in the HEAT repeat is to assist or orchestrate centromere/kinetochore segregation and spindle assembly checkpoint (SAC) control. In addition, we found that mitotically arrested cold-sensitive mutants *dis1-203* and *dis2-11* were rescued when they were combined with the *cnd3-L269P* mutant at 22°C (Fig. 5F), indicating that the aberrant mitotic arrest in *dis1-203* and *dis2-11* probably due to activation of SAC, was compromised by mutation of *cnd3-L269P*.

### Rescue of non-SMC ts mutants by histone deacetylase inhibitor TSA

**Fig 6:**
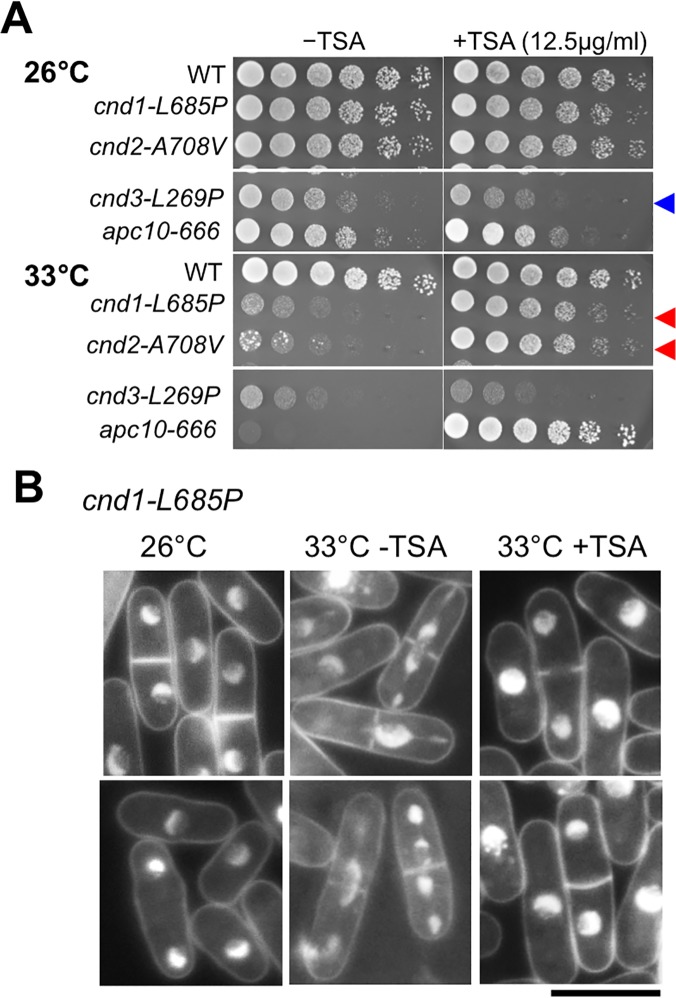
Non-SMC mutants, *cnd1-L685P* and *cnd2-A708V*, are rescued by trichostatin A, a histone deacetylase inhibitor. **A**. *cnd1-L685P* and *cnd2-A708V* are ts and are unable to form colonies at 33°C, a semi-permissive temperature, but they form normal colonies in the presence of trichostatin A (TSA), an inhibitor of histone deacetylase. *cnd3-L269P*, on the other hand, was slightly hyper-sensitive to TSA at 26°C. The mutant *apc10-666* is the control strain that was strongly rescued by TSA at 33°C [58]. **B**. Consistent with the results above, mitotic defects of *cnd1-L685P* (33°C in the absence of TSA for 5 hrs) were not observed in the culture at 33°C in the presence of 12.5μg/ml TSA for 5hrs (right panel). The bar is 10 μm.

A group of histone deacetylase (HDAC) enzymes are involved in heterochromatin formation, which is important for centromere function; therefore, we tested *cnd3-L269P* sensitivity to trichostatin A (TSA, an HDAC inhibitor). Indeed *cnd3-L269P* was sensitive to TSA (blue arrowhead in Fig. 6A), while other mutants, which were not TBZ sensitive, were not sensitive to TSA. Interestingly, *cnd1-L685P* and *cnd2-A708V*, which are ts, but not damage-sensitive, were actually rescued by 12.5 μg/mL TSA (red arrowheads in Fig. 6A). The mitotic mis-segregation phenotype of *cnd1-L685P* at 33°C was suppressed in the presence of TSA (Fig. 6B). These unexpected rescuing or hypersensitive phenotypes (in the presence of TSA) suggested that condensin non-SMC mutations might interact with centromeric proteins such as Mis18, a loader of CENP-A histone [57], APC (anaphase promoting complex/cyclosome), securin, separase, and cohesin loader Mis4. Kimata et al [58] showed that mutants of APC (*apc10-666*) and separase-securin, *cut1* and *cut2* are rescued by TSA. In contrast, Δ*mad2*-deletion, *mis4-242*, *mis6-302*, Δ*hos2* and *slp1-362* were sensitive to TSA.

### Increased DNA content from a haploid to a diploid level in six non-SMC mutants

During the course of genetic analysis, we observed that spores from 6 of 21 strains did not germinate, when they were crossed with the wild-type haploid (*h* ^+^ *leu1*). Tetrads were analyzed: Four spores formed, but they were not viable. The six strains were *cnd1-N914Y*, -*M951R*, -*G984R*, *cnd3-C101R*, -*S546R*, and -*D584G*. Five were HEAT mutants. The remaining mutant, *cnd1-N914Y*, resides close to one HEAT (H4) repeat. None of the mutants involved proline substitution, suggesting that the effects of these mutations on protein conformation might not be severe. Four of five HEAT mutations changed from residues with small side chains to the bulky arginine, causing significant conformation perturbation. Two of six showed the ts phenotype, while the majority (5/6) were damage-sensitive (either Ts^-^ Ds^-^ or Ds^-^). The inviable spore phenotype was observed at 26°C for all six strains above.

**Fig 7:**
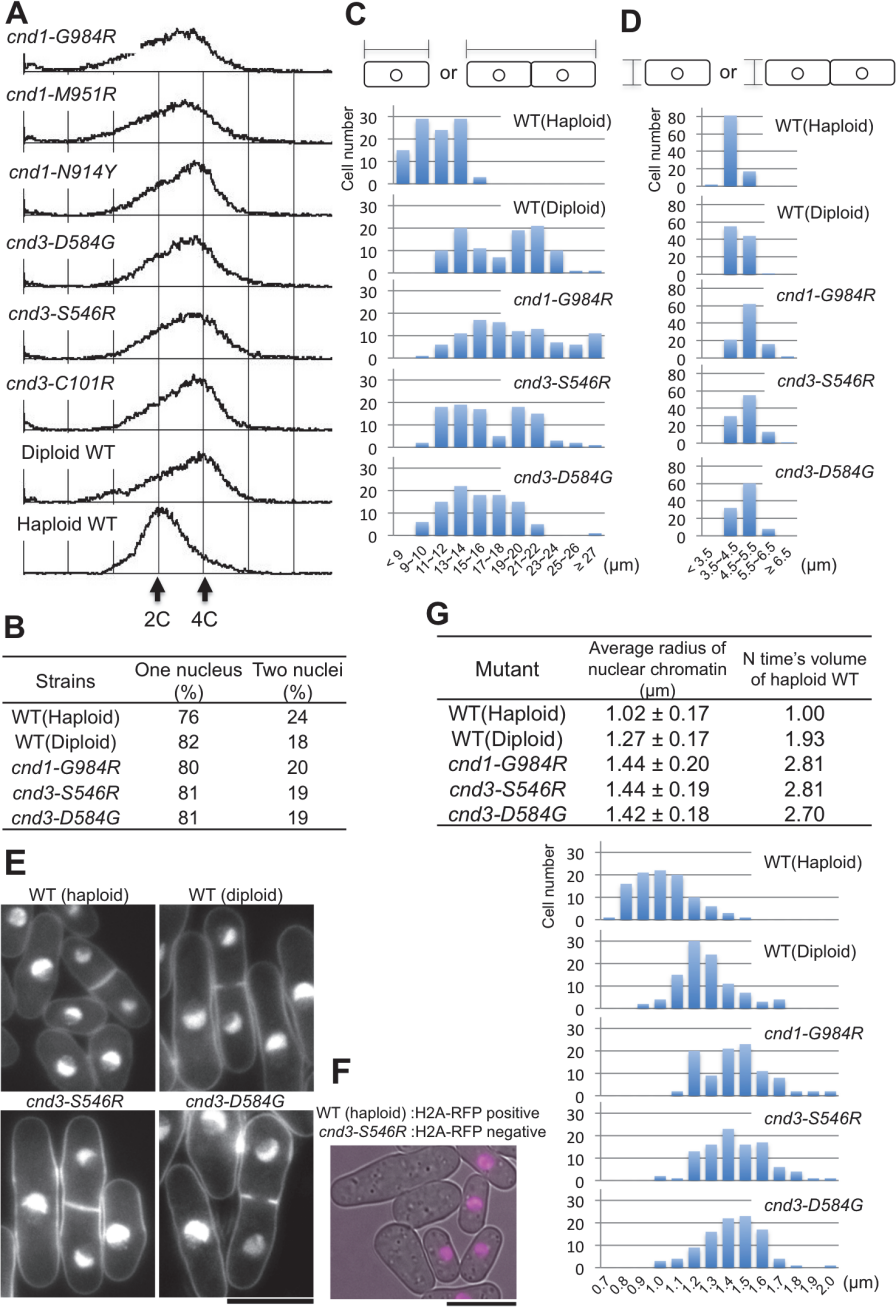
Damage-sensitive, non-SMC mutants are impaired in ploidy maintenance. **A**. FACS data of wild type (haploid, diploid), and six mutants. See text. **B**. The number of nuclei per cell in the wild-type (haploid, diploid) and three mutant cells. About 80% of all cells contained a single nucleus, while the remaining 20% contained two nuclei with or without the septum. The latter cells represent those after nuclear division and prior to cytokinesis in the cell division cycle. See top figures in **C**. **C**. Cell length distributions of wild type (haploid, diploid), and three non-SMC *cnd* mutants. Diploid cells and mutant cells are longer than haploid cells. **D**. Cell width measurements of wild types (haploid and diploid) and mutant cells. **E**. Micrographs of wild types (haploid and diploid) and two mutant cells stained with DAPI are shown. The bar indicates 10 μm. **F**. Equal numbers of histone H2A-RFP haploid wild-type cells and *cnd3-S546R* mutant cells from liquid cultures were mixed together for microscopy. All small cells are RFP positive, while large cells are RFP negative. The bar indicates 10 μm. **G**. Average nuclear radius (with standard deviations) was measured for wild type (haploid, diploid), and three mutant cells. Three mutant nuclei were clearly larger than that of the wild-type haploid. The top panel indicates the average length. The bottom panel shows distributions of nuclear radii.

We performed fluorescence-activated cell sorting (FACS) analysis (Experimental procedures). *S*. *pombe* wild-type and mutant cells were cultured and when they were growing exponentially at 26°C, cells were harvested, and prepared for FACS analysis (Materials and Methods) (Fig. 7A). Unexpectedly, the amount of DNA in these six mutants was found to be basically equal to that of diploid cells (4C) (approximately 80% of growing *S*. *pombe* cells are in G2 phase). Because the majority (∼80%) of these mutant cells contained single nuclei (Fig. 7B), similar to values for wild-type haploid (2C) and diploid (4C DNA) cells, binuclear cells were unlikely for these mutant strains.

Cells grown exponentially at 26°C were then stained with DAPI and observed using fluorescence microscopy. Cells were often elongated and cell width exceeded that of wild-type cells. Quantitative cell length and width data for 100 cells in each of three strains that increased DNA content are shown with wild-type haploid and diploid controls (Fig. 7C and D). DAPI-stained micrographs of two strains are shown in Fig. 7E. To confirm that these mutant cells are definitively larger than the wild-type haploid cells, we employed the wild-type haploid expressing red RFP-tagged histone H2A, which was cultured independently and then mixed with *cnd3-S546R* cells that did not contain RFP. Only small cells showed the H2A-RFP positive nucleus (Fig. 7F). This result with the internal control firmly demonstrated that *cnd3-S546R* mutant cells were bigger than haploid wild type.

We found that the amount of DAPI-stained mutant nuclear chromatin was also greater than that of control wild-type cells, when cells were grown at 26°C. The average size of the nuclear chromatin region and size distributions for each of the three strains are summarized in the Table of Fig. 7G, top and bottom panels, respectively. The size was greater than that of wild-type haploid nuclear chromatin, even bigger than that of diploid nuclear chromatin. Three mutants revealed a volume of nuclear chromatin roughly 2.7–2.8-fold bigger than that of control wild-type haploid cells. In contrast, other *cnd* mutants cells with haploid DNA content were not big, and their cell sizes resembled those of wild-type haploids.

## Discussion

Condensin plays a variety of roles in chromosome dynamics, but its genetic dissection has been hampered due to the lack of mutants that cause defects in its diverse and unrecognized functions. Previous mutant screening of *S*. *pombe* SMC and non-SMC mutants [17, 44–46] was restricted, as only cytological assays were employed to identify phenotypes with impaired chromosome condensation and segregation. In the present study, screening was targeted to obtain mutants in the three non-SMC subunit genes, assayed comprehensively (e.g., ts phenotype or drug-sensitivity assay). By such means, we were able to isolate and characterize 21 distinct, single-amino acid substitution mutants (8 *cnd1*, 1 *cnd2* and 12 *cnd3)* for the three non-SMC subunit genes, the isolation procedures of which are described in detail in Fig. 2A. These mutants may cover diverse roles of condensin non-SMC subunits, and shed light on functions hitherto unknown for non-SMC proteins. The role of non-SMC subunits has been scarcely understood in any organism.

Sixteen mutants (5 *cnd1*, 1 *cnd2* and 10 *cnd3*) produced the ts phenotype. At the restrictive temperature (36°C), these mutant cells failed in chromosome segregation and condensation in mitosis. The ts phenotype was indistinguishable from that of SMC ts mutants of *S*. *pombe* [12]. This supports the notion that severe functional loss of any of the three non-SMC subunits results in failure of holocondensin’s essential mitotic function. In contrast, five mutants (3 *cnd1*, 2 *cnd3*) produced a novel, non-ts, non-mitotic phenotype, but are sensitive to HU, CPT and/or UV. Curiously, none of them is proline substituted with the exception of *cnd1-L1037P*. Four of the five non-ts mutations reside in HEAT repeats. Defects in this group of damage-sensitive mutations are apparently relatively mild so that mutant holocondensin may retain its mitotic function.

**Fig 8:**
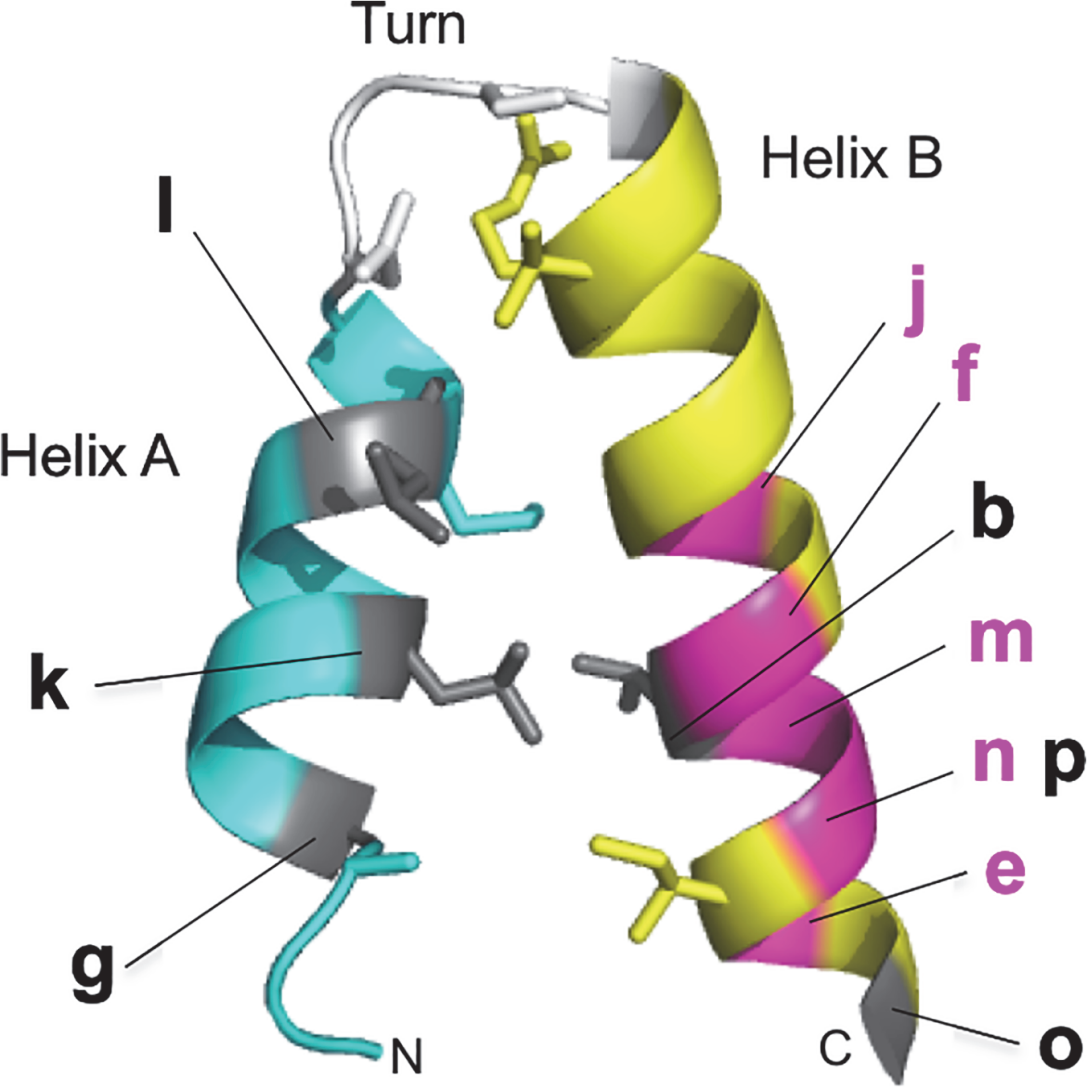
Summary of *cnd1* and *cnd3* HEAT mutant locations. Localizations of all HEAT mutants identified in this study are shown in one HEAT unit. See text.

Eleven mutations reside in HEAT repeats. Their locations are assembled in one repeat (Fig. 8; b, e, f and g, for Cnd1, j, k, l, m, n, o and p for Cnd3) (see also Fig. 3). None of them is located in the turn. The majority (8/11) are found in helix B, suggesting that helix B might play a key role in Cnd1 and Cnd3 proteins. Indeed, five mutants with doubled DNA content were found in helix B (Fig. 8, magenta). The remaining *cnd1-N914Y* did not occur in the HEAT repeat, but was situated close to the amino-terminus of HEAT 4. Three mutations in helix A (*cnd1-L1037P*, *cnd3-L126P*, *cnd3-L269P*) are DNA damage-sensitive at the permissive temperature, but they are small haploid-like cells. They are all proline substitutions. One possible explanation is that helices A and B interact with different protein species. Alternatively, the roles of helix A and B may be distinct in determining the 3D conformation. It would be interesting to introduce the same mutations in higher eukaryotic homologs and to study the phenotype if it does not cause lethal phenotypes. Known mutations in condensin non-SMC genes either cause disease, such as human microcephaly (MCPH1, [20]; NCAPG2, [59], or bovine body size increase [21, 22].

A striking mutant phenotype obtained in this study is that cells of six mutants (*cnd1-N914Y*, -*M951R*, -*G984R*, *cnd3-C101R*, -*S546R and D584G*) were larger than and contained twice the DNA of wild-type haploids (from 2C to 4C), suggesting that these mutant cells maintained a diploid-like state. DNA contents were measured for cells dividing normally at 26°C. When crossed with wild-type haploids, most spores formed were inviable, consistent with the notion that these mutant cells behave like diploid cells. Mutation sites of these mutants are either within or close to HEAT repeats. Interestingly, none of the mutations involved proline, and the majority involved arginine substitutions (4/6). We suppose that arginine replacement may not perturb protein conformation as much as proline substitution. Alternatively, in changing to a diploid-like state, a particular change in protein-protein interaction might be required in these mutants.

Our results are consistent with the notion that these mutant cells behave like diploid cells, (4C DNA in G2 phase). Meiosis between haploid wild type and these mutant cells was abnormal, resulting in barely viable spores. Massive aneuploidy seemed to have occurred due to the triploid meiosis between haploid and diploid [60]. Certain fission yeast genes are implicated in diploidization. The *cdc2-tws1* mutation [60, 61], *cut8-563* [62], and *myo2* [63] are known to cause diplodization. How condensin *cnd1* and *cnd3* mutations cause a diploid-like state remains to be investigated. Because meiosis was not involved in generating these mutations, defects in meiosis II are unlikely. Diploidization of *cut8-563* occurs at the restrictive temperature (36°C), and 60–70% of the cells surviving after two generations are diploid. Cut8 is located in the nuclear envelope and tethers proper localization of 26S proteasome along the nuclear membrane [64, 65]. The nuclear envelope architecture might be implicated in diploidization, since the volume of the nucleus should be related to maximal and minimal DNA content in cells. Non-SMC *cnd1* and *cnd3* mutations seemed to have more chromatin than wild-type diploid nuclei. These mutations might have caused the expansion of nuclear and chromosome scaffold (e.g., [66]), which might accompany the increase of DNA content. Very recently it was shown that nuclear architecture and nuclear size of human cells in interphase are disrupted by RNAi depletion of condensin SMC and non-SMC subunits [67]. The increased nuclear size is consistent with the present results.

Phenotypes produced by *cnd3-L269P* are of considerable interest regarding the link between Cnd3/Ycg1/CAP-G and mitotic regulation involving kinetochores/centromeres and spindle assembly checkpoint (SAC). *S*. *pombe* GFP-tagged condensin was previously shown to be localized at centromeres/kinetochores during mitosis [68]. The mitotic phenotype of *cnd3-L269P* mutants in the presence of TBZ showed large and small nuclei and unequal segregation of cen2-GFP, which are hallmarks of centromeric mutants. Cnd3 might directly contribute to the kinetochore function of condensin.

Mad2, located at mitotic kinetochores, is required for the spindle assembly checkpoint (SAC), and Δ*mad2* deletion mutants are sensitive to TBZ [53]. The strong synthetic phenotype of *cnd3-L269P Δmad2* double mutants is consistent with the hypothesis that Cnd3 may directly or indirectly interact with Mad2 for proper arrangement of mitotic kinetochores. Mad2 was reported to physically interact with centromere protein Mis6 [69]. The additive interaction between *cnd3*-*L269P* and centromeric mutant *mis6-302* is also consistent with the notion that Cnd3 plays an important role in the interaction between condensin and kinetochore. Dis1 and Mtc1/Alp14 (a homolog of human TOG, frog XMAP215 and budding yeast Stu2) implicated in proper chromosome segregation, interact with kinetochore microtubules and contain HEAT repeats [38, 41, 70]. We show in this study that the cold-sensitive phenotype of *dis1-203* (similar to Mtc1/Alp14) and *dis2-11 (*type 1 protein phosphatase catalytic subunit) mutants is partly rescued by *cnd3-L269P*, while Δ*mtc1/alp14* mutant interacts negatively. Dis1 and Mtc1/Alp14 appear to function in an opposing way toward *cnd3-L269P* at the kinetochore during mitosis. To our knowledge, only one TBZ-sensitive condensin mutant was isolated recently [71]. Two types of condensin, I and II, exist in higher eukaryotes, while fungi, such as budding yeast and fission yeast contains only one condensin. Present and previous results show that fungal condensin does interact with centromeres/kinetochores perhaps through Cnd3/Ycg1/CAP-G, one of three non-SMC subunits.

In summary, we isolated 21 fission yeast mutants defective in condensin’s three non-SMC subunits. Beside previously known mitotic functions in chromosome condensation and segregation and in DNA repair, we identified two novel condensin functions. First, HEAT repeat-containing subunits, Cnd1 and Cnd3, are involved in maintaining the haploid genome state. Each of the three *cnd1* and the three *cnd3* mutations resided in or near the HEAT repeats caused an increase in cell size and doubled DNA content. It remains to be determined whether the size increase of nucleus is the primary event caused by these diploid HEAT-repeat mutations. These mutants normally proliferate, but do not form viable spores in meiotic segregation when crossed with wild-type haploids, probably due to triploid meiosis producing massive aneuploidy. Condensin thus seems to be required for maintaining the ploidy level via its HEAT-containing subunits. Secondly, we show that one *cnd3-L269P* mutant is defective in centromere/kinetochore function and spindle assembly checkpoint control. Consistently, this mutant, bearing an altered residue in the conserved HEAT repeat is hypersensitive to TBZ, a tubulin poison. These results together demonstrate novel aspects of the role of non-SMC, HEAT-containing subunits of condensin. The non-SMC condensin subunits Cnd1 and Cnd3 may play an unexpected role in cell growth and/or ploidy level stability.

## Materials and Methods

### Media, strains and plasmids

YPD (1% yeast extract, 2% polypeptone, 2% D-glucose) and Edinburgh Minimal Medium 2 (EMM2) were used for culturing *S*. *pombe*, and MEA medium was used for sporulation [72, 73]. The *S*. *pombe* haploid wild-type strain 972 *h* ^-^ and its derivative mutant strains, including the temperature-sensitive (ts) *cut14-208*, *cut3-477*, *cut14-Y1*, *mis6-302*, and *apc10-666*, and cold-sensitive (cs) *dis1-203* and *dis2-11* were used [12, 16, 50, 58, 74]. Deletion mutants of the *mad2* ^+^ and *mtc1* ^+^ genes, and RFP-tagged histone H2A strain were described previously [40, 75, 76]. For construction of integration plasmids into the endogenous *cnd* ^*+*^ gene loci, entire *cnd* ^+^ ORF sequences and their downstream sequences (∼500bp long) were amplified independently, and were then ligated into pBluescript containing an antibiotic marker (pBS-hygR for *cnd1* ^+^ and *cnd3* ^+^, pBS-kanR for *cnd2* ^+^). Cells were counted using a hematology analyzer (Sysmex FDA-500).

### Error-prone PCR for mutagenesis

Error-prone PCR was performed as described previously [47]. Briefly, the DNA fragment containing the *cnd* ^+^ gene, the hygR/kanR marker and a 500 bp downstream sequence was amplified from integration plasmids by PCR under error-prone conditions: The reaction buffer used contained 0.8 mM dNTP mixture (equimolar solution of dATP, dCTP, dGTP and dTTP), 8 mM Mg^2+^, 2 μM forward primer, 2 μM reverse primer, TaKaRa Ex Taq HS: 1.25 U/ 50 μL. PCR was repeated cycling of three steps for 30 cycles: denature DNA at 98°C for 10 sec, primer annealing at 50°C for 30 sec and extension at 72°C for 6 min.

### Screening of temperature-sensitive and/or damage-sensitive mutants

Amplified DNA fragments of *cnd1*, *cnd2*, and *cnd3* prepared by error-prone PCRs were used for transformation of the *S*. *pombe* haploid wild-type strain 972 *h* ^–^. Hygromycin (or G418 for *cnd2*)-resistant clones were picked up. Transformants were plated at 26°C, 36°C in the absence of DNA-damaging reagents, 26°C in 2mM hydroxyurea (HU), 26°C in 7.5μM camptothecin (CPT) and 26°C after 150J/m^2^ ultraviolet irradiation (UV) for 3 days. Colony formation was compared with that of the control culture at 26°C in the absence of DNA damage. Ts^-^ and damage-sensitive (Ds^-^) mutants were selected. Mutation sites in isolated strains were determined by Sanger dideoxy sequencing of the genomic *cnd* regions after PCR amplification. Twenty-one strains contained single, non-synonymous mutations. Subsequently, mutations were introduced into the wild-type strain by site-directed mutagenesis as described previously [75] and phenotypes produced were examined for reproducibility. Briefly, complementary pairs of oligonucleotide DNAs with mutations were used as primers, followed by two rounds of PCR. Chromosome integration into endogenous *cnd* ^*+*^ loci was followed, and the resulting integrant strains were tested for ts and/or damage sensitive phenotypes.

### Microscopy

Each ts mutant strain was first grown in YPD medium at the permissive temperature (26°C), and then shifted to the restrictive temperature (36°C). DAPI staining was performed as described previously [77], and cells were observed with a BZ9000 microscope (Keyence, Japan). To visualize localization of centromere 2 (cen2), the *cnd3-L269P* mutant strain was crossed with the GFP-tagged cen2-GFP strain [51]. DAPI-stained nuclear chromatin and cen2-GFP centromere signals were observed with the BZ9000. Cell length and width were measured using the “Between 2-points” tool of the “Measurement module” in BZ-II Analyzer software (Keyence, Japan). Radii of nuclei were measured using the “Radius” tool in the software.

### FACScan analysis

Procedures described [78, 79] were followed to determine the average nuclear DNA content. Haploid and diploid wild-type cells were used as controls. Cells (1 × 10^7^) were stained with propidium iodide (final concentration, 12.5 μg/ml), digested with RNase I, and analyzed with a FACScan (Beckton-Dickinson).

## Supporting Information

S1 Fig DAPI-stained mutants cells (Fig. 4A) cultured at 36°C for 5 h. After 5 h at 36°C, dead cells due to abnormal cytokinesis which displayed strong fluorescence of DAPI due to absorption to the cell wall materials were observed. Hence the states of missegregated chromosomes could not be seen.
(TIF)

S2 Fig Temperature sensitivity of the three *cnd* ts mutants in Fig. 4B and C. *S*. *pombe* wild-type (WT), and *cnd* mutant cells were spotted on solid agar plates and cultured at various temperatures (26°C, 30°C, 33°C and 36°C).
(TIF)

S1 Table Summary of initial-screening for *S*. *pombe* condensin non-SMC mutants. At Step 6 (Fig. 2A), after streaking a large number of transformants and nucleotide sequencing of 130 strains, we obtained a total of 77 *S*. *pombe* chromosome integrant strains, which contained single or multiple mutations in the *cnd1*, *cnd2*, or *cnd3* gene and displayed either Ts^-^, Ts^-^Ds^-^, or Ds^-^ phenotype.
(TIF)
