# Supplementary figures and images for: Condensin HEAT Subunits Required for DNA Repair, Kinetochore/Centromere Function and Ploidy Maintenance in Fission Yeast
Source: PLoS One. 2015 Mar 12;10(3):e0119347. doi: 10.1371/journal.pone.0119347 (PMC4357468; doi:10.1371/journal.pone.0119347)

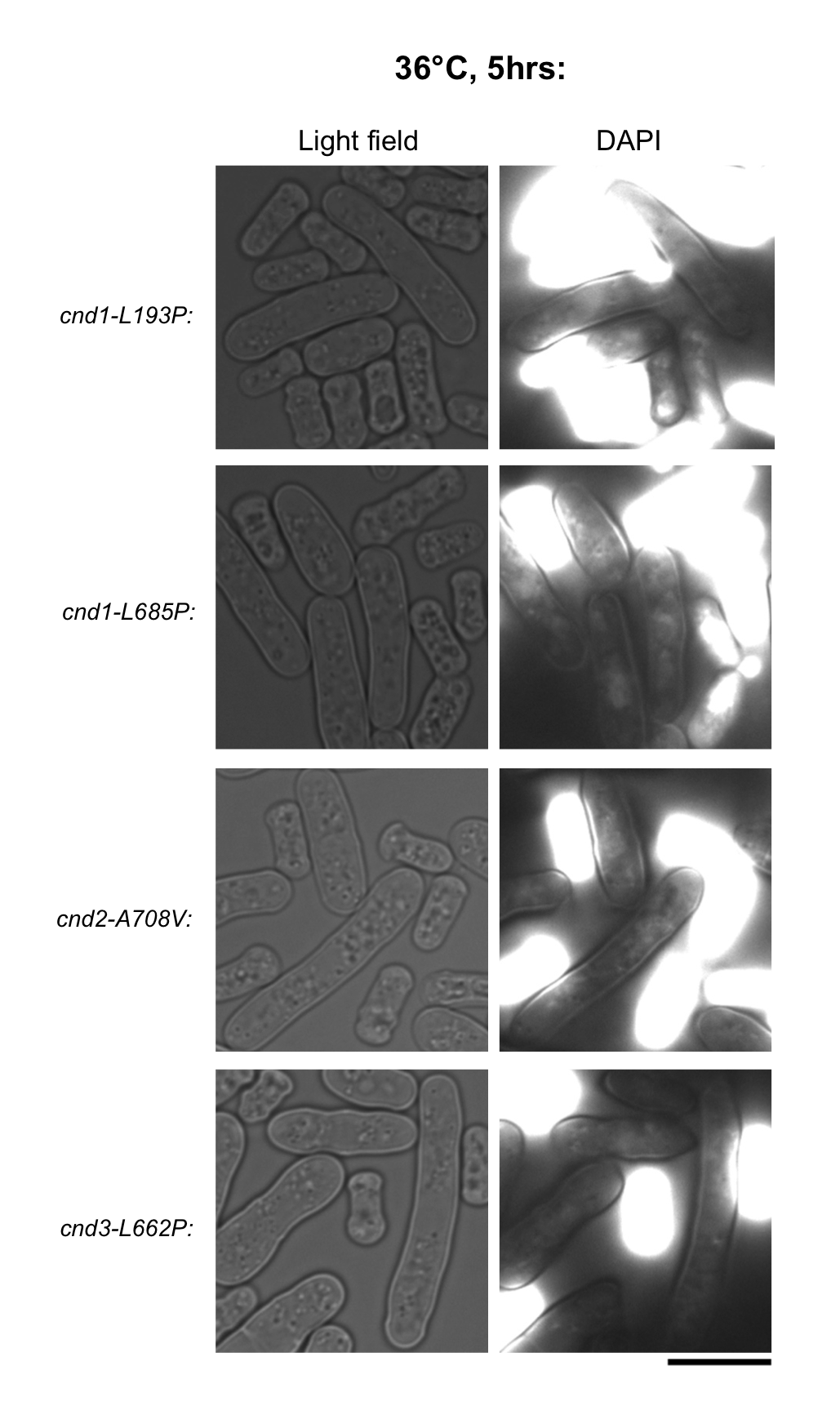

Supplement: S1 Fig — After 5 h at 36°C, dead cells due to abnormal cytokinesis which displayed strong fluorescence of DAPI due to absorption to the cell wall materials were observed. Hence the states of missegregated chromosomes could not be seen. (TIF) [file pone.0119347.s001.tif]

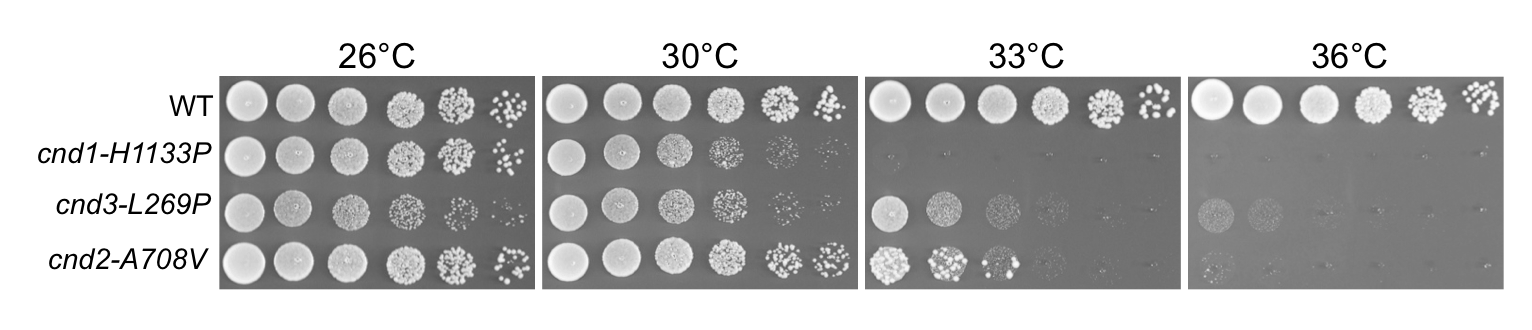

Supplement: S2 Fig — S. pombe wild-type (WT), and cnd mutant cells were spotted on solid agar plates and cultured at various temperatures (26°C, 30°C, 33°C and 36°C). (TIF) [file pone.0119347.s002.tif]

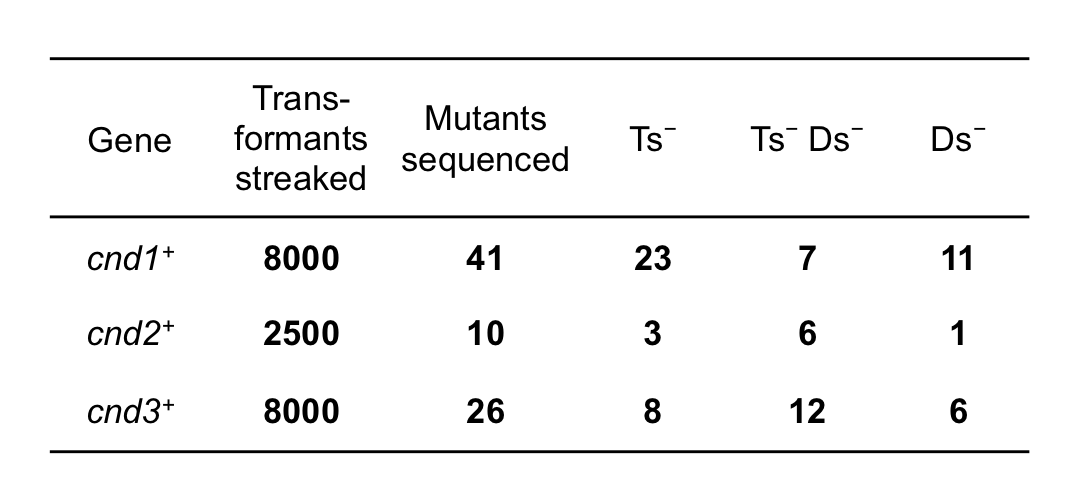

Supplement: S1 Table — At Step 6 (Fig. 2A), after streaking a large number of transformants and nucleotide sequencing of 130 strains, we obtained a total of 77 S. pombe chromosome integrant strains, which contained single or multiple mutations in the cnd1, cnd2, or cnd3 gene and displayed either Ts-, Ts-Ds-, or Ds- phenotype. (TIF) [file pone.0119347.s003.tif]
